# Ex Vivo Generation of CAR Macrophages from Hematopoietic Stem and Progenitor Cells for Use in Cancer Therapy

**DOI:** 10.3390/cells11060994

**Published:** 2022-03-15

**Authors:** Daniela Paasch, Johann Meyer, Andriana Stamopoulou, Daniela Lenz, Johannes Kuehle, Doreen Kloos, Theresa Buchegger, Astrid Holzinger, Christine S. Falk, Christina Kloth, Constantin S. von Kaisenberg, Hinrich Abken, Axel Schambach, Nico Lachmann, Michael Morgan, Thomas Moritz

**Affiliations:** 1 Research Group Reprogramming and Gene Therapy, Hannover Medical School, 30625 Hannover, Germany; paasch.daniela@mh-hannover.de (D.P.); stamopoulou.andriana@mh-hannover.de (A.S.); sirus22@gmx.de (C.K.); 2 Institute of Experimental Hematology, Hannover Medical School, 30625 Hannover, Germany; johann.meyer@uni-oldenburg.de (J.M.); daniela.lenz@helmholtz-hzi.de (D.L.); kloos.doreen@mh-hannover.de (D.K.); buchegger.theresa@mh-hannover.de (T.B.); schambach.axel@mh-hannover.de (A.S.); lachmann.nico@mh-hannover.de (N.L.); 3 REBIRTH Research Center for Translational and Regenerative Medicine, Hannover Medical School, 30625 Hannover, Germany; 4 Center for Molecular Medicine Cologne, University of Cologne, 50931 Cologne, Germany; johanneskuehle@gmx.de; 5 Department of Pediatric Pneumology, Allergology and Neonatology, Hannover Medical School, 30625 Hannover, Germany; 6 Leibniz Institute for Immunotherapy, Division of Genetic Immunotherapy, University Regensburg, 93053 Regensburg, Germany; astrid.holzinger@klinik.uni-regensburg.de (A.H.); hinrich.abken@klinik.uni-regensburg.de (H.A.); 7 Institute of Transplant Immunology, Hannover Medical School, 30265 Hannover, Germany; falk.christine@mh-hannover.de; 8 Department of Obstetrics and Gynecology, Hannover Medical School, 30625 Hannover, Germany; vonkaisenberg.constantin@mh-hannover.de; 9 Division of Hematology/Oncology, Boston Children’s Hospital, Harvard Medical School, Boston, MA 02115, USA

**Keywords:** chimeric antigen receptors, macrophages, cancer, hematopoietic stem cells, solid tumors, cancer immunotherapy, phagocytosis

## Abstract

Chimeric antigen receptor (CAR) T-cell therapies have shown impressive results in patients with hematological malignancies; however, little success has been achieved in the treatment of solid tumors. Recently, macrophages (MΦs) were identified as an additional candidate for the CAR approach, and initial proof of concept studies using peripheral blood-derived monocytes showed antigen-redirected activation of CAR MΦs. However, some patients may not be suitable for monocyte-apheresis, and prior cancer treatment regimens may negatively affect immune cell number and functionality. To address this problem, we here introduce primary human hematopoietic stem and progenitor cells (HSPCs) as a cell source to generate functional CAR MΦs ex vivo. Our data showed successful CAR expression in cord blood (CB)-derived HSPCs, with considerable cell expansion during differentiation to CAR MΦs. HSPC-derived MΦs showed typical MΦ morphology, phenotype, and basic anti-bacterial functionality. CAR MΦs targeting the carcinoembryonic antigen (CEA) and containing either a DAP12- or a CD3ζ-derived signaling domain showed antigen redirected activation as they secreted pro-inflammatory cytokines specifically upon contact with CEA^+^ target cells. In addition, CD3ζ-expressing CAR MΦs exhibited significantly enhanced phagocytosis of CEA^+^ HT1080 cells. Our data establish human HSPCs as a suitable cell source to generate functional CAR MΦs and further support the use of CAR MΦs in the context of solid tumor therapy.

## 1. Introduction

Adoptive cell therapy (ACT) represents a promising approach to support standard cancer treatment strategies, including surgery, radiation, and chemotherapy [1]. Efforts to strengthen the patient’s immune system through administration of cellular immune therapeutics have resulted in significant improvements for cancer patients [2]. In particular, the chimeric antigen receptor (CAR) T cell therapy has recently revolutionized the fight against hematological malignancies [3,4,5]. CARs are artificial receptors that consist of an extracellular antigen-binding unit, e.g., derived from a single chain antibody fragment (scFv), a hinge region, a transmembrane sequence, and an intracellular signaling domain responsible for specific cell activation. CAR principles were also applied to other immune cell types, such as natural killer (NK) cells, which showed encouraging results in first clinical trials in patients with CD19^+^ lymphoid tumors [6]. However, successful use of CAR T and CAR NK cells against solid tumors remains to be fully established, and strategies to improve the efficacy and persistence of CAR T and CAR NK cells are currently being explored [7].

Another immune effector cell type potentially suitable for CAR cell therapy are MΦs, since they have a high phagocytic capacity [8] and are antigen-presenting cells [9]. MΦs are able to infiltrate tumors [10], actively attack cancer cells, and simultaneously orchestrate important immune responses and activate bystander cells in order to enhance the anti-tumor response. Furthermore, MΦs are capable of cytokine secretion [11] and exhibit a high loading capacity, which can be exploited for use of these cells as cargo vehicles [12]. More than 30 years ago, MΦs were used as adoptive cell therapies against solid tumors, and autologous transfer of blood-derived monocytes was shown to be safe [13]. However, efficient anti-tumor responses were not achieved [14]. This led to the hypothesis that the full anti-cancer potential could only be realized via the addition of a cell-activating stimulus. Evidence for the potential use of immune therapeutic approaches employing genetically engineered MΦs was demonstrated in pre-clinical cancer models by promoting an anti-tumor immune response via activation of additional immune cells and counteracting the immunosuppressive tumor microenvironment, resulting in inhibition of tumor progression [15,16,17,18,19].

Morrissey and colleagues recently expanded the approach of genetically engineered MΦs and equipped murine bone marrow (BM)-derived MΦs with CARs that contained different signaling domains known to increase phagocytosis [20]. These so-called ‘CAR-P macrophages’ exhibited significantly enhanced phagocytosis of CD19-labeled beads as well as cancer cells, which led to reduced growth of CD19^+^ lymphoma cells. In addition, Klichinsky and colleagues genetically engineered human CD14^+^ monocytes with CARs using an adenoviral approach and differentiated them into MΦs ex vivo. They showed that these CAR MΦs displayed increased phagocytic activity and anti-cancer responses against CD19^+^ hematological malignancies as well as HER2^+^ or mesothelin^+^ solid tumors [21]. A potential drawback for CAR MΦ generation from autologous monocytes, however, is the fact, that in cancer patients, the amount and functionality of circulating monocytes may be impacted by previous therapy, thus preventing adequate monocyte-apheresis [22,23,24].

To overcome this limitation, we suggest primary human hematopoietic stem and progenitor cells (HSPCs) as an additional cell source to generate CAR MΦs. Such HSPCs can be derived from BM, peripheral blood (PB) after G-CSF mobilization, or cord blood (CB), and extensive banks of HLA-typed donors have been established for these sources [25,26]. For proof-of-concept studies, we generated CAR MΦs from CB-HSPCs to target tumor cells that express the carcinoembryonic antigen (CEA). CEA is a cell adhesion protein that is highly expressed in various solid tumors including colorectal, gastric, and pancreatic cancers, and is a well-investigated tumor-associated antigen (TAA) [27]. After transduction of CD34^+^ CB-HSPCs and differentiation to MΦs, we show that the generated CAR MΦs exhibited typical MΦ phenotype, morphology, and basic anti-bacterial phagocytic function. The generated CAR MΦs exhibited the capacity to redirect their phagocytic activity and cytokine secretion upon contact with CEA-expressing cancer cells, suggesting their suitability for use in cancer therapy. 

## 2. Materials and Methods

### 2.1. Cell Line Culture Conditions

MONO-MAC-6 and THP-1 cells were cultured in 12-well suspension plates using RPMI 1640 medium (GIBCO, Life Technologies, Carlsbad, CA, USA) supplemented with 10% (*v*/*v*) fetal bovine serum (FBS; Sigma Aldrich, St. Louis, MO, USA) and 1% (*v*/*v*) penicillin/streptomycin (P/S; PAA Laboratories, Pasching, Austria). For cultivation of MONO-MAC-6 cells, 10 µg/mL human insulin (Sigma-Aldrich, St. Louis, MO, USA) was added to the medium. HT1080 cells were cultured in 6-well adherent plates using Dulbecco’s modified Eagle’s medium (DMEM; Invitrogen, Waltham, MA, USA) supplemented with 10% (*v*/*v*) FBS and 1% (*v*/*v*) P/S. All cell lines were split twice a week in a 1:20 ratio and cultured under standard humidified conditions at 37 °C and 5% CO_2_. All cell lines were purchased from the German Collection of Microorganisms and Cell Cultures GmbH (DSMZ, Braunschweig, Germany) and routinely tested for mycoplasma.

### 2.2. Differentiation of THP-1 Cells to MΦs

To differentiate THP-1 cells into MΦs, 5 × 10^5^ cells were seeded in a 12-well suspension plate in 2 mL supplemented RPMI 1640 medium and treated with 5 ng/mL of phorbol 12-myristate-12 acetate (PMA; Sigma-Aldrich, St. Louis, MO, USA) for 48 h. Afterwards, the cells were centrifuged at 300× *g* for 5 min at room temperature (RT) and transferred into a new well of a 12-well plate with fresh RPMI 1640 medium without PMA and cultured for an additional 24 h.

### 2.3. Generation of Lentiviral CAR Constructs and Viral Vector Production

The αCEA-IgG1-2B4-DAP12 CAR vector (in the following referred to as DAP12 CAR) was inserted into a third-generation self-inactivating lentiviral backbone (pRRL.cPPT.CAG) using the *AgeI* and *SalI* restriction sites. To allow visualization of modified cells, an eGFP reporter sequence was introduced into the SalI site of the pRRL.cPPT.CAG_DAP12 CAR vector. The αCEA-IgG1-CD28-CD3ζ CAR vector [28] (in the following referred to as CD3ζ CAR) was cloned into the same lentiviral backbone as the DAP12 CAR. The final CAR vectors contain a humanized αCEA scFv and human sequences for the other CAR domains and were verified by DNA sequencing (GATC, Constance, Germany). A CAG-driven eGFP control vector was provided by Dr. Malte Sgodda. HT1080 cells were engineered to artificially express CEA. An mCherry-expressing vector (LeGO-C2 plasmid) was kindly provided by Prof. Boris Fehse (UKE Hamburg).

The production of lentiviral vector particles was performed using HEK293T cells, which were cultured in DMEM containing 10% (*v*/*v*) FBS, 1% (*v*/*v*) P/S, 20 mM HEPES (PAA Laboratories, Pasching, Austria), and 25 μM chloroquine (Sigma-Aldrich, St. Louis, MO, USA). For the transfection of HEK293T cells, the calcium phosphate method was performed using 8 μg/mL of pcDNA3.GP.4xCTE (gag/pol), 5 μg/mL of pRSV-Rev, 5 μg/mL of lentiviral vector plasmid, and 2 μg/mL of vesicular stomatitis virus glycoprotein (VSVg) plasmid pMD.G. At 24 h and 48 h post-transfection, the viral supernatants were harvested, using 0.2 µM pore size filters, and concentrated by centrifugation using an Avanti J-26XP centrifuge (Beckman Coulter, Brea, CA, USA) overnight (o.n) at 10,000× *g* at 4 °C. To determine lentiviral vector titers, an established protocol was used. In short: 1 × 10^5^ adherent SC-1 cells were transduced with a defined volume of viral particles in a dilution series of 1:100, 1:1000, and 1:10,000 using cell culture medium supplemented with protamine sulfate (4 µg/mL) [29]. Transduction efficiency was analyzed 72 h post-transduction for eGFP and 96 h post-transduction for mCherry viral titers using a CytoFLEX S flow cytometer (Beckman Coulter, Brea, CA, USA).

### 2.4. Transduction of Cell Lines with Lentiviral Constructs

A total of 2 × 10^5^ cells were transduced with a multiplicity of infection (MOI) of 2 using the respective culture medium containing 4 μg/mL of protamine sulfate. Viral transduction was performed for 24 h, prior to washing and retransferring the cells back to standard culture medium. Transduction efficiency was analyzed 72 h post-transduction for eGFP vectors and 96 h for mCherry vectors using flow cytometry.

### 2.5. Isolation and Cultivation of Human CD34^+^ Cells from Umbilical Cord Blood

In cooperation with the Department of Gynecology and Prenatal Medicine (Hannover Medical School), umbilical cord blood (UCB) was collected from healthy donors after obtaining signed informed consent. The study was conducted according to the guidelines of the Declaration of Helsinki, and informed consent was obtained from all subjects involved in the study prior to UCB collection, as approved by the Hannover Medical School Ethics Committee (protocol code 1303-2012). All samples were anonymized. In short, peripheral blood mononuclear cells (PBMCs) were isolated under Ficoll density gradient centrifugation (400× *g* for 45 min at RT). Red cell lysis was performed for 3 min at RT (buffer: 4.15 g NH_4_Cl; 2.3 g KHCO_3_; 0.5 mL 0.5 M EDTA; in 500 mL H_2_O), and CD34^+^ cells were enriched from PBMCs by magnetic separation using a bead-conjugated anti-CD34 antibody (Miltenyi Biotec, Bergisch Gladbach, Germany), according to the manufacturer’s instructions. Isolated CD34^+^ cells were cultured in StemSpan medium (STEMCELL Technologies, Vancouver, BC, Canada) supplemented with 2 mM L-glutamine, 1 mM P/S (both Thermo Fisher Scientific, Waltham, MA, USA), 100 ng/mL human Stem Cell Factor (hSCF), 100 ng/mL human Fms-related Tyrosine Kinase 3 Ligand (hFLT3L), and 50 ng/mL human thrombopoietin (hTPO) (all from Peprotech, Hamburg, Germany) at 37 °C and 5% CO_2_.

### 2.6. Transduction of CD34^+^ Cells and Differentiation into MΦs

At 24 h after isolation from cord blood, 2 × 10^5^ cells were transduced with an MOI of 5 using the supplemented StemSpan culture medium containing 4 μg/mL of protamine sulfate and 1% (*v*/*v*) Poloxamer Synperonic F108 (Sigma-Aldrich, St. Louis, MI, USA). After 24 h, the cells were washed and retransferred to standard culture medium. Transduction efficiency was analyzed 72 h post-transduction via flow-cytometric analysis of eGFP expression. After sorting for eGFP expression using a FACSAria Fusion flow cytometer (BD Biosciences, Franklin Lakes, NJ, USA) on day 5 post-transduction, the cells were cultured in standard CD34^+^ cell culture medium for an additional 3 days. On day 8, in order to start the differentiation towards MΦs, CD34^+^ cells were transferred to RPMI 1640 medium containing 10% (*v*/*v*) FBS and 1% (*v*/*v*) P/S, 100 ng/mL hM-CSF, 100 ng/mL hGM-CSF, and 20 ng/mL hIL-3 for 5 days. Subsequently, cells were cultured in RPMI 1640 medium containing 10% (*v*/*v*) FBS, 1% (*v*/*v*) P/S, and 100 ng/mL hM-CSF for 5 more days. 

### 2.7. Cytospins

A total of 4 × 10^4^ cells were resuspended in 150 μL of phosphate-buffered saline (PBS) and centrifuged on glass slides using a Cytofuge^®^ (Medite, Burgdorf, Germany) at 700× *g* for 10 min. Afterwards, May–Grünwald Giemsa staining was performed. In short, cells were stained in 0.25% (*w*/*v*) May–Grünwald solution (Roth, Karlsruhe, Germany) for 5 min, washed in distilled water and stained with 1:20 diluted GIEMSA solution (Roth) for 20 min. After a second wash step, the slides were dried overnight and embedded in Roti-Histokitt mounting solution (Roth, Karlsruhe, Germany). Pictures were taken using an Olympus IX71 with the CellSens Dimension imaging software (Olympus, Shinjuku, Japan). 

### 2.8. Phagocytosis Assay (pHrodo)

To determine basic anti-bacterial phagocytic activity, 1.5 × 10^5^ cells were incubated with 10 µL of pHrodo-coupled *E.coli* particles (#P35361 Life Technologies, Carlsbad, CA, USA) in phenol red-free RPMI 1640 medium, supplemented with 10% (*v*/*v*) FBS, 1% (*v*/*v*) P/S, 20 mM HEPES, and 100 ng/mL hM-CSF. After 2 h, pictures were taken using an Olympus IX71 fluorescence microscope (Olympus, Shinjuku, Japan) with the Cellsens Dimension software, and quantitative analysis was performed by flow cytometry.

### 2.9. Flow Cytometry

Surface marker and reporter gene expression was determined using a CytoFLEX S flow cytometer (Beckman Coulter, Brea, CA, USA). The following antibodies were used to characterize cells: hCD45-PE-Cy7, hCD11b-APC, hCD14-PE, hCD163-APC, hCD86-PE, hCD16-PE-Cy7 (all from eBioscience, Frankfurt am Main, Germany), hHLA-DR-APC, hCD206-PE-Cy7 (all from Biolegend, Fell, Germany), hIgG-PE (SouthernBiotech, Birmingham, AL, USA), and isotype control mouse-IgG1κ (eBioscience, Frankfurt am Main, Germany). Except for hIgG-PE staining, the cells were blocked for 5 min at RT with human Fc-Block TruStain FcX^TM^ (Biolegend, Fell, Germany), prior to antibody staining, according to the manufacturer’s instructions. For all depicted flow cytometric analyses, at least 50,000 events were acquired per sample. Subsequent analysis was performed using FlowJo10 (BD Biosciences, Franklin Lakes, NJ, USA).

### 2.10. Quantification of Vector Copy Number

Integrated vector copy numbers (VCN) were determined using TaqMan technology. Genomic DNA was isolated from sorted CD34^+^ cells that were further differentiated into MΦs using the GeneElute Mammalian Genomic DNA Miniprep Kit (Sigma-Aldrich, St. Louis, MO, USA). Quantitative real-time polymerase chain reaction (qPCR) was performed on a StepOnePlus light cycler (Applied Biosystems, Waltham, MA, USA). For absolute quantification of integrated vector copies, a standard curve of serial dilutions of plasmid standard harboring the genomic housekeeping gene Polypyrimidine Tract Binding Protein 2 (*PTBP2*) sequence was used (10^6^, 10^5^, 10^4^, and 10^3^ copies/µL). The following primers and probes were utilized to detect the integrated lentiviral vector: Woodchuck Hepatitis Virus Posttranscriptional Regulatory Element (*wPRE*) primers (forward: GAGGAGTTGTGGCCCGTTGT; reverse: TGACAGGTGGTGG-CAATGCC) and probe (5′-FAM-CTGTGTTTGCTGACGCAAC-3′-BHQ1); *PTBP2* primers (forward: TCTCCATTCCC-TATGTTCATGC; reverse: GTTCCCGCAGAATGGTGAGGTG) and probe (5′-JOE-ATGTTCCTCGGACCAACTTG-3′-BHQ1).

### 2.11. CAR MΦ Stimulation with the Anti-Idiotypic Anti-BW2064 Antibody

For antibody-mediated CAR stimulation, wells of a standard 96-well plate for suspension culture (Sarstedt, Nümbrecht, Germany) were coated with 50 µL of 5 µg/mL of the anti-idiotypic antibody BW2064 (provided by Dr. Astrid Holzinger, RCI Regensburg) that is specific for the anti-CEA scFv of the CAR [30] diluted in PBS and kept overnight at 4 °C. The coated wells were washed twice with PBS and blocked with 1% bovine serum albumin (BSA) diluted in PBS for 30 min at RT. The PBS was discarded, and 5 × 10^4^ CD34^+^ cell-derived MΦs were seeded using RPMI 1640 culture medium supplemented with 100 ng/mL human Macrophage Colony-Stimulating Factor (hM-CSF). After 24 h of stimulation, the supernatant was collected, centrifuged for 5 min at 300× *g*, and stored at −20 °C. 

### 2.12. Quantification of MΦ Phagocytic Activity against HT1080 Target Cells by Flow Cytometry

To assess phagocytosis against HT1080 cells, 2 × 10^5^ CD34^+^ cell-derived MΦs were seeded in an adherent 12-well plate and allowed to attach overnight. The seeded MΦs were stimulated with 500 ng/mL lipopolysaccharide (LPS, Sigma-Aldrich, St. Louis, MO, USA) for 3 h prior to the addition of 2 × 10^5^ WT/mCherry^+^ or CEA^+^/mCherry^+^ HT1080 cells. After 4 h, supernatants were collected and filtered through a 70 µM pore size filter into a FACS tube. Wells were washed once with PBS and incubated with 500 µL Accutase for 20 min at 37 °C. The detached cells in the well were collected and remaining cells were washed once with cold PBS to ensure that all cells were collected into the flow cytometry tube. The cell mix was centrifuged at 300× *g* for 5 min and analyzed using a CytoFLEX S flow cytometer. Phagocytic events were identified by gating on single cells to exclude potential false-positive signals. The MΦs were then selected by eGFP expression, and the percentage of phagocytic events was determined on the basis of the mCherry signal within the gated eGFP^+^ population.

### 2.13. Confocal Microscopy and Quantitative Analysis of mCherry Expression

A total of 2 × 10^5^ CD34^+^ cell-derived MΦs were seeded onto a 12 mm round coverslip in 500 µL of RPMI 1640 culture medium supplemented with 100 ng/mL of hM-CSF and allowed to attach o.n. The seeded MΦs were stimulated with 500 ng/mL LPS (Sigma Aldrich, St. Louis, MO, USA) for 3 h prior to the addition of 2 × 10^5^ WT/mCherry^+^ or CEA^+^/mCherry^+^ HT1080 cells. After 4 h, the supernatant was discarded, and each well was carefully washed twice with 500 μL of PBS. Cells were fixed with 500 μL of 4% paraformaldehyde (PFA) (Sigma-Aldrich, St. Louis, MO, USA diluted in PBS) for 15 min and washed 3× with 500 μL of 1× tris-buffered saline (TBS) for 5 min. TBS was removed and the coverslips were allowed to dry. After 5 min, the coverslips were carefully placed in an inverted orientation onto a glass slide and embedded using ProLong Gold Antifade mounting medium (Life Technologies, Carlsbad, CA, USA). The coverslips were allowed to dry o.n. All images were taken using an inverted Leica TCS SP8 microscope (Leica Microsystems, Wetzlar, Germany) with the LAS X analysis software. Subsequent quantification of mCherry signal within the eGFP^+^ MΦ population was determined using ImageJ.

### 2.14. Cytokine Secretion

For co-culture experiments, 5 × 10^4^ CD34^+^ cell-derived MΦs or THP-1-derived MΦs were seeded in adherent 96-well tissue culture plates o.n. The cells were then stimulated with 500 ng/mL LPS for 3 h followed by the addition of 5 × 10^4^ WT or CEA^+^ HT1080 cells. After 4 h, supernatants were collected, centrifuged at 300× *g* for 5 min, and analyzed using the human IL-6 and TNFα ELISA Ready-Set-Go! Kit (R&D Systems, Minneapolis, MN, USA) according to the manufacturer’s instructions. The supernatants from anti-idiotypic antibody stimulation (Section 2.11) were analyzed using the same kits. Two biological replicates were analyzed for basal cytokine/chemokine secretion pattern using a Bio-Plex Pro Human Cytokine 27-plex Assay (Bio-Rad, Hercules, CA, USA).

### 2.15. Viability and Apoptosis Assay

For the determination of viable and apoptotic THP-1 cells upon PMA-treatment, the cells were washed with PBS and harvested using 1 mL Accutase for 10 min at 37 °C. The cells were stained at RT for 20 min using 2 µL of an Annexin-V-APC antibody (Biolegend, San Diego, CA, USA), washed again with PBS, and centrifuged for 5 min at 300× *g*. Quantitative analysis was performed directly after the addition of 1 µL of PI, using a CytoFlex S flow cytometer.

### 2.16. Statistical Analysis

Statistical analyses were performed using Prism 9.0 software (GraphPad Software, San Diego, CA, USA). Unless otherwise mentioned, one-way ANOVA with respective Tukey’s post hoc testing was performed for statistical comparison between groups. Error bars throughout the paper denote 95% confidence intervals of the mean. **** indicates *p* < 0.0001, *** indicates *p* < 0.001, ** indicates *p* < 0.01, and * indicates *p* < 0.03.

## 3. Results

### 3.1. Validation of Anti-CEA CAR Vectors in Myeloid Cell Lines

**Figure 1:**
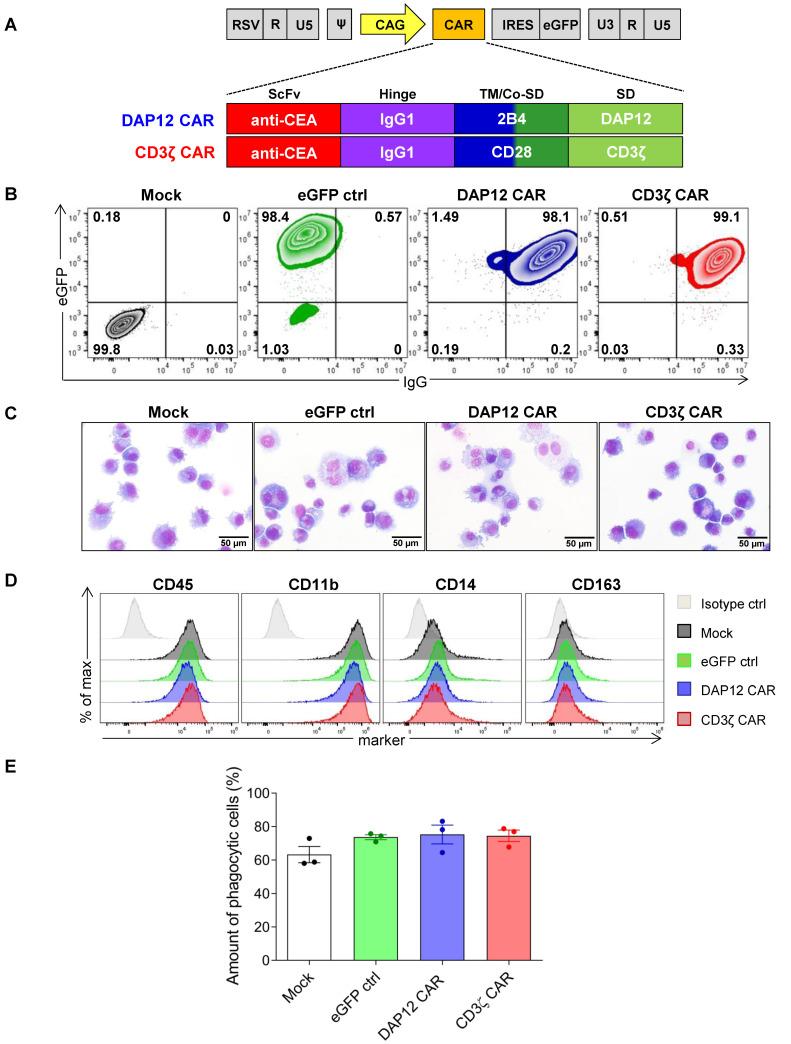
Expression of anti-CEA CARs in differentiated CAR THP-1 cells. (**A**) Scheme depicting the structure of both anti-CEA CARs. (**B**) Flow cytometric analyses of eGFP and CAR expression in differentiated THP-1 cells. Controls included mock-transduced THP-1 cells (Mock) and THP-1 cells transduced with a lentiviral vector designed to express eGFP alone (eGFP ctrl). Plots are shown for a representative out of 3 experiments. (**C**) Microscopic analyses of THP-1 cell morphology using May–Grünwald-stained cytospins (40× magnification using the fluorescence microscope Olympus IX71, scale bar: 50 µM). (**D**) Flow cytometric analyses of surface markers on differentiated THP-1 cells. Data are representative out of 2 experiments. (**E**) Analysis of phagocytic activity of *E. coli* particles in THP-1 cells as revealed by flow cytometry. Median phagocytosis percentages with standard deviations are shown (*n* = 3). scFv: single chain variable fragment; TM: transmembrane domain; Co-SD: costimulatory signaling domain; IgG: human IgG1 CH2CH3; ctrl: control.

To generate CAR macrophages (CAR MΦs) from human hematopoietic stem and progenitor cells (HSPCs), we utilized two different CARs, both designed to target the carcinoembryonic antigen (CEA). CARs were identical with regard to the antigen recognition domain and the extracellular IgG1 hinge region but incorporated different intracellular signaling domains (Figure 1A). One CAR contained T cell-specific domains with transmembrane and costimulatory domains derived from CD28 sequences and CD3ζ as the signaling domain (CD3ζ CAR), as CD3ζ has been shown to be functional in the context of CAR MΦs designed to target other tumor-associated antigens (TAA) (20,21). The second CAR contained MΦ-specific domains with transmembrane and costimulatory domains derived from 2B4 sequences and DAP12 as the signaling domain (DAP12 CAR), given that DAP12 plays an important role in the innate immune response by inducing a pro-inflammatory immune reaction. Moreover, incorporation of DAP12 has been shown to activate CAR T and CAR NK cells following contact to their respective tumor antigens [31,32]; however, it has thus far not been utilized in the context of CAR MΦs. For both constructs, CAR expression is driven by a CAG promoter and coupled to an eGFP reporter gene via an internal ribosomal entry site (IRES). 

**Figure A1:**
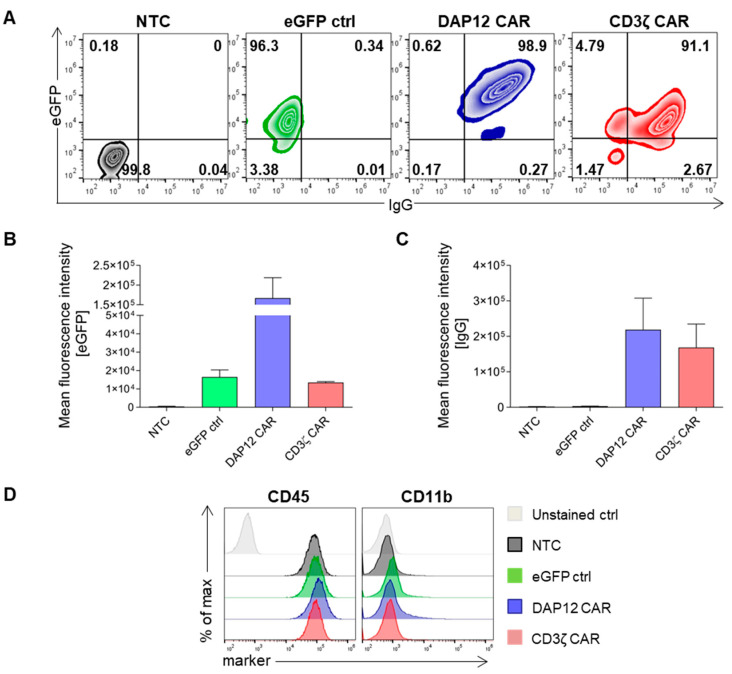
Assessment of CAR expression and characterization in MONO-MAC-6 cells. (**A**) Representative flow cytometric analyses of eGFP and CAR expression in MONO-MAC-6 cells transduced with eGFP alone (eGFP ctrl), the DAP12 CAR construct, or the CD3ζ CAR construct. Mock-transduced cells (mock) were used as additional controls. Mean fluorescence intensity of (**B**) eGFP and (**C**) IgG expression in MONO-MAC-6 cells. Data (**A**–**C**) are represented as the mean ± s.e.m. of *n* = 2 biological replicates. (**D**) Flow cytometric analyses of MΦ surface markers (*n* = 1).

As a first step to assess CAR expression in myeloid cells, we modified the human monocytic cell line MONO-MAC-6 by lentiviral transduction with both anti-CEA CAR constructs or with a control vector expressing only eGFP. Flow cytometric analyses of CAR and eGFP expression showed a double positive population for both CAR constructs. While DAP12 CAR-expressing MONO-MAC-6 cells showed a stronger eGFP expression, both anti-CEA CAR constructs exhibited similar levels of CAR expression as assessed by flow cytometric detection of the IgG1 hinge region (Figure A1A–C). No major effect of anti-CEA CAR expression on CD45 or CD11b surface marker expression was observed for MONO-MAC-6 cells (Figure A1D).

**Figure A2:**
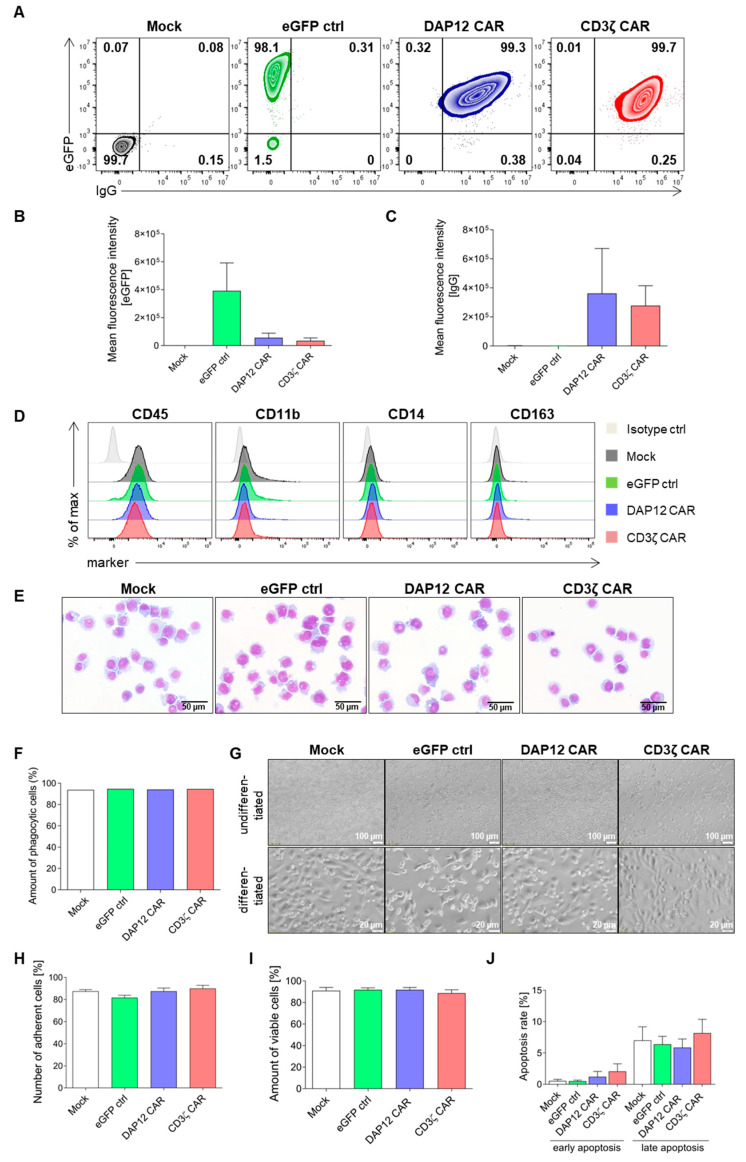
Characterization of THP-1 cells transduced with CAR constructs. (**A**) Representative flow cytometric analyses of eGFP and CAR expression in THP-1 cells. Mean fluorescence intensity of (**B**) eGFP and (**C**) IgG expression in THP-1 cells. Data are represented as the mean ± s.e.m. of *n* = 3 biological replicates. (**D**) Flow cytometric analyses of monocytic surface markers on unmodified (mock) and modified THP-1 cells (*n* = 2). (**E**) Microscopic analyses of THP-1 cell morphology using May–Grünwald-stained cytospins (40× magnification using the fluorescence microscope Olympus IX71, scale bar: 50 µM). (**F**) pHrodo-based analysis of phagocytic cells via flow cytometry (*n* = 1). (**G**) Microscopic images of undifferentiated and PMA-differentiated THP-1 cells using the fluorescence microscope Olympus IX71 (scale bar: 100 µm and 20 µm). (**H**) Analysis of THP-1 differentiation efficiency shown by percentage of adherent cells after PMA stimulation. Flow cytometric analysis of (**I**) viability and (**J**) apoptosis rate in unmodified and modified THP-1 cells. Data shown in (**H**–**J**) are represented as the mean ± s.e.m. of *n* = 3 biological replicates. ctrl: control.

To test the effects of the CAR constructs in a second human monocytic cell line, we also transduced THP-1 cells with the CAR constructs. Again, flow cytometric analyses showed strong co-expression of the CARs and eGFP in transduced cells for both anti-CEA constructs (Figure A2A–C). CAR-modified cells demonstrated similar surface marker (CD45^+^, CD11b^+^, CD14^+^, and CD163^-^) expression patterns (Figure A2D) and typical monocyte morphology when compared to the mock-transduced or eGFP-expressing control cells (Figure A2E). To assess a potential effect of CAR expression on THP-1 functionality, the basic anti-bacterial phagocytic capability was assessed by a pHrodo assay to measure the phagocytosis of *E. coli* bacteria. This analysis showed similar levels of phagocytic activity under all conditions tested, regardless of the CAR expression (Figure A2F), indicating the conserved functional capacities of engineered cells. 

The THP-1 cell line represents a commonly used cell line model to mimic primary human monocytes/MΦs; upon stimulation with phorbol 12-myristate 13-acetate (PMA), THP-1 cells differentiate towards a MΦ-like phenotype [33]. We used these cells to analyze potential effects of CAR expression on MΦ morphology, phenotype, and basic functionality. Importantly, CAR expression did not impair the differentiation of THP-1 cells into MΦ-like cells, as shown by the similar morphology and percentages of cell adherence upon PMA stimulation (Figure A2G,H). Furthermore, cultures of CAR-expressing cells had similar viability (Figure A2I) and apoptosis rates compared to mock-transduced or eGFP only-expressing control cell cultures (Figure A2J). The differentiated, CAR-modified THP-1 cells again demonstrated strong co-expression of eGFP and the CAR (>98% double positive cells) with similar expression levels for the DAP12- and the CD3ζ-CAR constructs (Figure 1B). The same pattern of monocytic and MΦ-like morphology was detected for both anti-CEA constructs as well as the two control cultures (Figure 1C) after PMA-based MΦ differentiation. Induced differentiation of THP-1 cells increased CD11b, CD14, and CD163 expression (Figure 1D, compared to Figure A2E). The expression of these markers was similar for CAR-modified and control cells, indicating that the presence of the CAR did not impact the differentiation capacities. Similar levels of basic anti-bacterial phagocytic activity were also observed for control and CAR-modified cells (Figure 1E), indicating MΦ-like functional capacities. In summary, these data demonstrated successful CAR expression in two myeloid cell lines as well as THP-1-derived MΦ-like cells without major effects on cell phenotype, morphology, or basic functions.

### 3.2. Differentiated CAR THP-1 Cells Showed Enhanced Cytokine Secretion upon Co-Culture with HT1080 Cells Engineered to Express CEA

**Figure 2:**
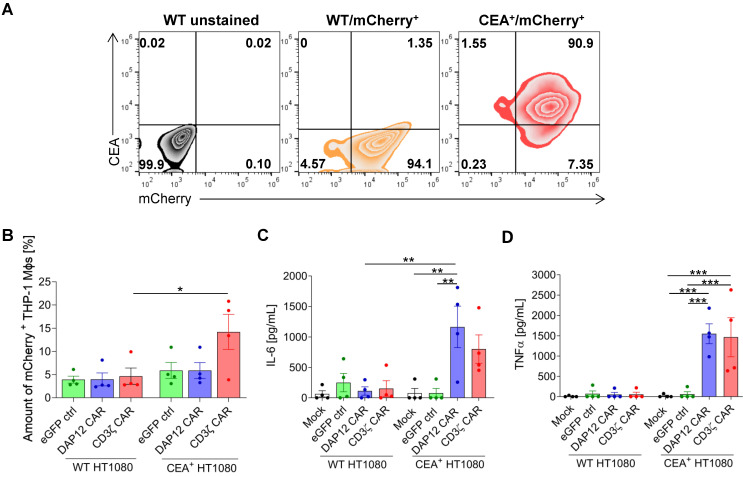
CAR-mediated cancer cell phagocytosis and cytokine secretion of THP-1-derived MΦs. (**A**) Flow cytometric analyses of mCherry and CEA expression in HT1080 target cells. (**B**) Percentage of mCherry^+^ MΦs after co-culture with target cells analyzed by flow cytometry (*n* = 4). ELISA assays showing (**C**) IL-6 and (**D**) TNFα secretion by THP-1-derived MΦs after co-culture with WT/mCherry^+^ or CEA^+^/mCherry^+^ HT1080 cells. Data (**C**,**D**) are represented as the mean ± s.e.m. of *n* = 4 biological replicates measured in duplicate. Statistical significance was calculated with one-way ANOVA using multiple comparisons (*** indicates *p* < 0.001, ** indicates *p* < 0.01, and * indicates *p* < 0.03). Mock: untransduced THP-1-derived MΦs, ctrl: control.

We next investigated the capability of differentiated anti-CEA-CAR THP-1 cells to target and phagocytose CEA-expressing cancer cells. To do so, we transduced wild-type (WT) and CEA^+^ HT1080 target cells with a monocistronic vector to additionally express mCherry as a reporter gene. These cells were named WT/mCherry^+^ HT080 and CEA^+^/mCherry^+^ HT1080. Flow cytometric analyses showed similar levels of mCherry expression for both target cells, whereas CEA expression was only detected in CEA-transduced HT1080 cells, as expected (Figure 2A). These cells were then used to investigate two main anti-cancer mechanisms of MΦs: (1) antigen-specific phagocytosis and (2) cytokine secretion upon co-culture with target cells. For analysis of phagocytic activity, GFP-labeled differentiated CAR THP-1 cells were co-cultured with WT/mCherry^+^ or CEA^+^/mCherry^+^ HT1080 cells and the relative mCherry signal inside the differentiated THP-1 cells was analyzed via flow cytometric analysis. After 4 h of co-culture using a 1:1 cell ratio, all THP-1-derived MΦ conditions showed an equivalent level of phagocytosis against WT/mCherry^+^ HT1080 cells (Figure 2B). In contrast, co-culture with CEA^+^/mCherry^+^ target cells revealed a significant increase of mCherry^+^ cells in CD3ζ CAR MΦs (averages of 14.2% ± 7.6% (CEA^+^/mCherry^+^ HT1080) vs. 4.7% ± 3.5% (WT/mCherry^+^), respectively). Cytokine secretion analysis showed a similar pattern. Here, analyses of secreted pro-inflammatory cytokines from these co-cultures revealed significantly enhanced secretion of IL-6 (Figure 2C) and an even stronger increase of TNFα secretion (Figure 2D) for both CAR constructs, specifically upon exposure of THP-1-derived MΦs to CEA^+^ HT1080 cells. To exclude unspecific activation or MΦ cross-activation by Fcγ receptors, monocultures of (transduced) MΦs were analyzed. No cytokine expression was detected in these controls (data not shown). These data indicate CEA-specific function by increased cytokine secretion for both anti-CEA-CARs as well as enhanced antigen-specific phagocytosis of target cells by CD3ζ CAR-expressing THP-1-derived MΦs.

### 3.3. CAR Expression Did Not Interfere with the Induced Differentiation of Cord Blood-Derived CD34^+^ Cells into Functional Macrophages

**Figure 3:**
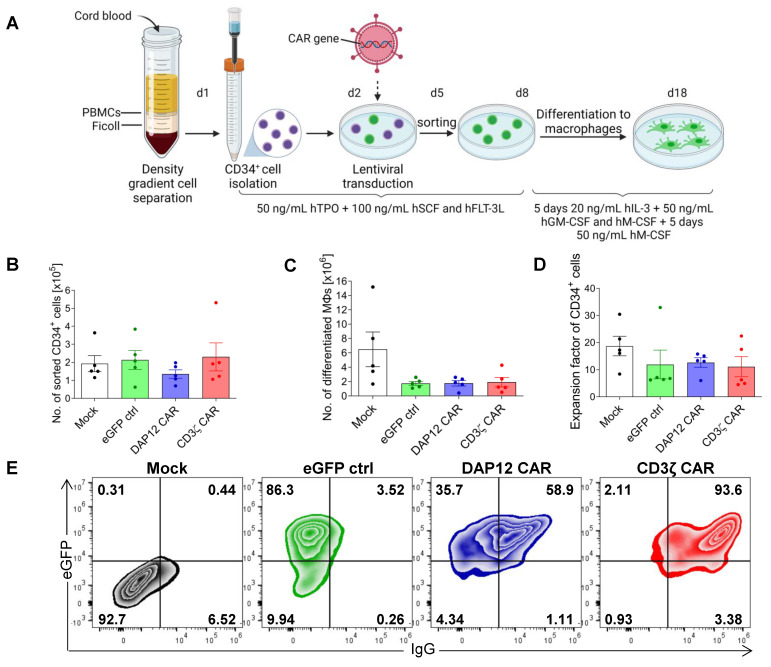

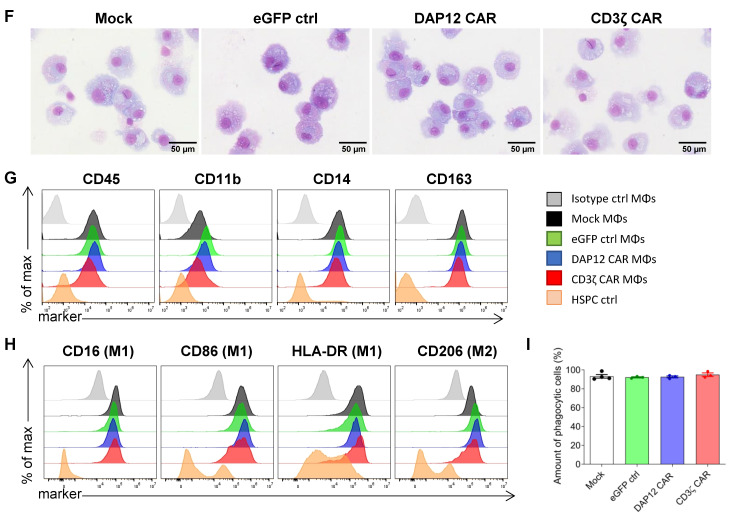
Generation and characterization of cord blood-derived anti-CEA CAR MΦs. (**A**) Overview of the CAR MΦ generation protocol showing the cord blood CD34^+^ cell isolation, transduction with CAR constructs, and differentiation into MΦs (created with BioRender.com). Numbers of (**B**) eGFP^+^ sorted CB-derived CD34^+^ cells and (**C**) differentiated MΦs obtained. (**D**) Expansion factor during differentiation of CB-derived CD34^+^ cells to MΦs. Cells were counted after sorting and at the end of the differentiation using a Neubauer chamber (*n* = 5). (**E**) Flow cytometric analyses of eGFP and CAR expression (as detected by the extracellular IgG CAR domain) in differentiated MΦs. Plots are shown for a representative out of 4 experiments. (**F**) Microscopic analysis of MΦ morphology in May–Grünwald-stained cytospins (40× magnification using the fluorescence microscope Olympus IX71, scale bar: 50 µm). (**G**) Flow cytometric analysis of MΦ surface markers. Plots are shown for a representative out of 4 experiments. (**H**) Flow cytometric analysis of MΦ polarization M1 and M2 surface markers in transduced (eGFP^+^) MΦs. Plots are shown for a representative out of 3 experiments. (**I**) pHrodo-based analysis of basic anti-bacterial phagocytic activity in MΦs by flow cytometry. Data are represented as the mean ± s.e.m. of *n* = 3–4 biological replicates. (**E**–**I**) Mock: non-transduced controls; ctrl: control; HSPC ctrl: hematopoietic stem and progenitor cell control.

**Figure A3:**
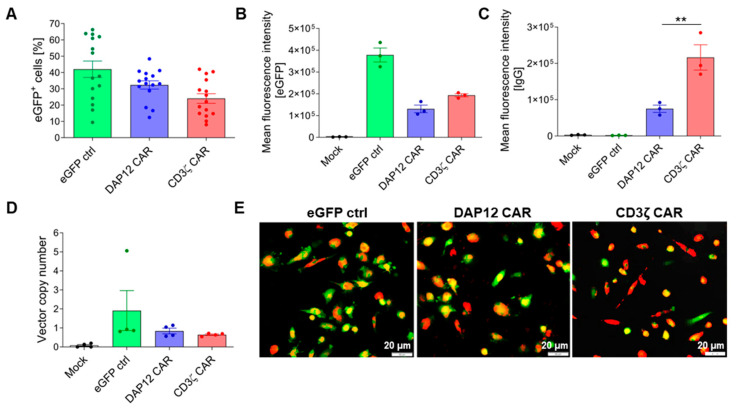
Validation of efficiency and MΦ characteristics after lentiviral transduction of HPSCs. (**A**) Transduction efficiencies in CD34^+^ cells using an MOI 5 (*n* = 15). Flow cytometric analysis of (**B**) eGFP and (**C**) CAR expression was determined in sorted and differentiated MΦs using an antibody to detect the IgG of the CAR constructs. Data are represented as the mean ± s.e.m. of *n* = 3 biological replicates. (**D**) Vector copy number analysis via qPCR. Data are represented as the mean ± s.e.m. of *n* = 4 biological replicates. (**E**) pHrodo-based analysis of phagocytic cells via fluorescent microscopy (20× magnification, scale bar: 20 µm). Statistical significance was calculated with one-way ANOVA using multiple comparisons (** indicates *p* < 0.01). Mock: untransduced CD34-derived MΦs; ctrl: control.

To evaluate CAR expression and function in MΦs derived from primary cells, CD34^+^ hematopoietic stem and progenitor cells (HSPCs) were isolated from cord blood samples; expanded in the stem cell-supporting medium StemSpan™ SFEM II supplemented with 50 ng/mL hTPO, 100 ng/mL hSCF, and 100 ng/mL hFLT3; and transduced with our CAR constructs (Figure 3A). Mean lentiviral transduction efficiencies in CD34^+^ cells were 42.0% for the eGFP ctrl (±19.5%), 32.0% for the DAP12 CAR (±9.9%), and 24.0% for the CD3ζ CAR (±11.4%) when an MOI of 5 was applied (Figure A3A). After transduction with the lentiviral vectors, the cells were sorted for eGFP expression (Figure 3B) and further expanded three days before being differentiated into MΦs via the use of 20 ng/mL hIL-3, 50 ng/mL hGM-CSF, and 50 ng/mL hM-CSF (Figure 3C). When the initial number of sorted CD34^+^ cells was compared to the amount of differentiated MΦs obtained after 18 days, a more than 10-fold increase was detected for all conditions with an overall lower expansion rate after lentiviral transduction compared to mock controls. However, CAR expression had no impact on the expansion rate, showing a similar level for both CAR constructs compared to eGFP ctrl-transduced MΦs (Mock: 17.66, eGFP ctrl: 11.91, DAP12 CAR: 12.6, and CD3ζ CAR: 11.13) (Figure 3D). After differentiation into MΦs, both CAR constructs maintained eGFP expression (Figure A3B) and showed CAR expression compared to mock- or eGFP-transduced MΦs, as shown by flow cytometric analysis of IgG stained MΦs (Figure 3C,E Lentiviral transduction led to a similar mean vector copy number (VCN) for the two CAR constructs (DAP12 CAR: 0.84 vs. CD3ζ CAR: 0.64) (Figure A3D). Typical MΦ morphology (Figure 3F) and surface marker (CD45^+^, CD11b^+^, CD14^+^, and CD163^+^) expression (Figure 3G) was observed for all mock-transduced and transduced cultures. Furthermore, the CAR MΦs showed the same heterogeneous polarization pattern, including M1 (CD16, CD86 and HLA-DR) and M2 (CD163, CD206) MΦ markers compared to controls (Figure 3H), revealing that the CAR expression per se does not affect the activation state of the cells. Similar levels of basic anti-bacterial phagocytic activity (more than 90% of cells) was observed for all mock-transduced and transduced cultures (Figure 3E,I), indicating that the basic function of these cells was maintained. These data demonstrated successful transduction of CB-derived HSPCs and differentiation into CAR MΦs with stable expression of CARs and no evidence that the CAR expression might alter CD34^+^ cell-derived MΦ phenotype, morphology, or basic anti-bacterial phagocytic function.

### 3.4. CD34^+^ Cell-Derived CAR MΦs Showed Cytokine Secretion upon CAR Stimulation

**Figure 4:**
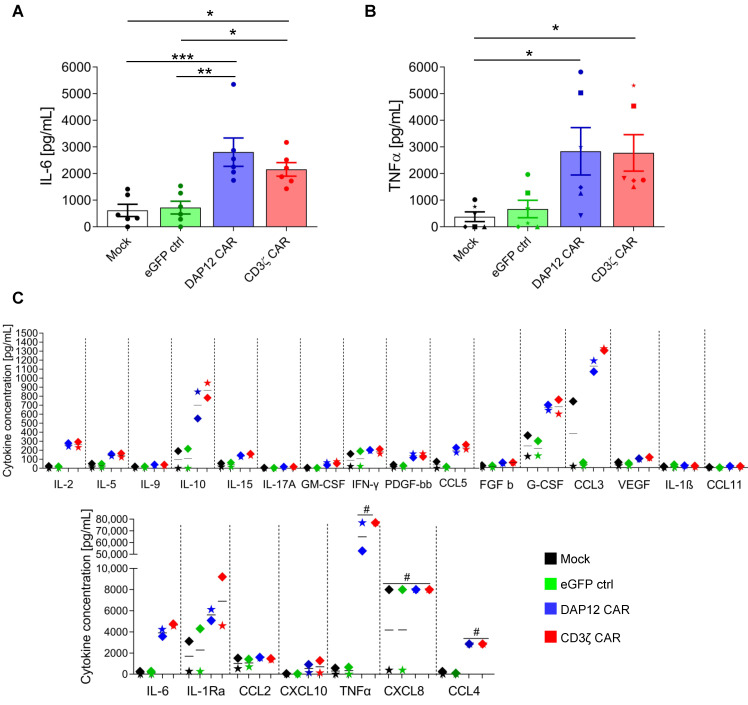
CAR-mediated cytokine secretion after stimulation of anti-CEA CAR MΦs with an anti-BW431/26 idiotypic antibody. ELISA assays exhibiting (**A**) IL-6 and (**B**) TNFα secretion from MΦs after 24 h stimulation with an immobilized anti-idiotypic antibody against the anti-CEA scFv. Data are represented as the mean ± s.e.m. of *n* = 6 biological replicates. Statistical significance was calculated with one-way ANOVA using multiple comparisons. (**C**) Two biological replicates (shown as colored boxes or stars) were further assessed for cytokine/chemokine production by a cytokine multiplex assay (*** indicates *p* < 0.001, ** indicates *p* < 0.01, and * indicates *p* < 0.03). Mock: untransduced CD34-derived MΦs; ctrl: control; #: value measured was above detection level.

Two different approaches were used to validate CAR function in CD34^+^ cell-derived MΦs. In the first approach, the anti-CEA CAR MΦs were stimulated with an immobilized anti-BW431/26 idiotypic antibody that was used as a surrogate antigen for CEA and was previously shown to specifically interact with the anti-CEA scFv used in the CAR constructs [34]. To exclude unspecific activation or MΦ cross-activation by Fcγ receptors, monocultures of (transduced) MΦs were analyzed. No cytokine expression was detected in these controls (data not shown); however, IL-6 (Figure 4A) and TNFα (Figure 4B) levels were markedly increased in the supernatant of anti-CEA CAR MΦs 24 h after stimulation with the anti-idiotypic antibody as a surrogate CAR antigen. These increases were significant compared to mock-transduced or eGFP controls and demonstrated specific functional activity of both our CAR constructs in MΦs. Next, the cytokine expression pattern of anti-CEA-CAR MΦs was further assessed using a cytokine multiplex array to analyze data from two biological replicates (Figure 4C). Following analysis, cytokines were categorized into three groups that showed (1) substantially increased secretion upon CAR activation (IL-10, G-CSF, CCL3, CCL4, IL-6, and TNFα), (2) moderately increased secretion upon CAR activation (IL-2, IL-5, IL-15, GM-CSF, IFN-γ, PDGF-bb, CCL5, VEGF, IL-1Ra, CXCL10, and CXCL8), or (3) no detected increase in secretion upon CAR activation (IL-9, IL-17, FGF b, IL-1ß, CCL11, and CCL2) compared to mock-transduced and eGFP control (ctrl) MΦs. Thus, the anti-BW431/26 idiotypic antibody induced antigen-specific activation of anti-CEA CAR MΦs, as shown by marked and broad-based cytokine secretion.

### 3.5. CD34^+^ Cell-Derived CAR MΦs Showed Enhanced Phagocytosis and Cytokine Secretion upon Contact with CEA^+^ HT1080 Cells

**Figure 5:**
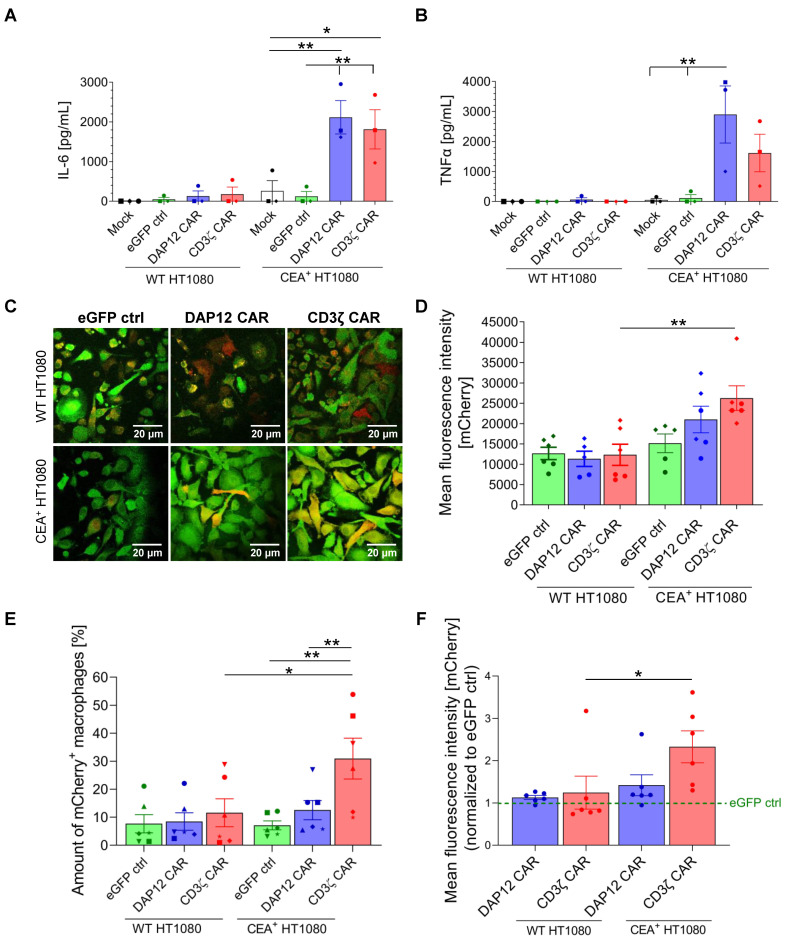
CAR-mediated cytokine secretion and phagocytosis after co-culture of MΦs with WT/mCherry^+^ or CEA^+^/mCherry^+^ HT1080 target cells. (**A**) IL-6 and (**B**) TNFα secretion in MΦs after co-culture with WT/mCherry^+^ or CEA^+^/mCherry^+^ HT1080 cells. Data are represented as the mean ± s.e.m. of *n* = 3 biological replicates. Statistical significance was calculated with one-way ANOVA using multiple comparisons. (**C**) Confocal microscopy images showing phagocytosis of WT/mCherry^+^ or CEA^+^/mCherry^+^ HT1080 cells (mCherry) by CAR MΦs (eGFP) (63× oil immersion objective of the Leica DMi8 confocal microscope, scale bar: 20 µm). Images are shown for a representative out of 2 experiments. (**D**) Analysis of confocal images depicted via the geometric mean of mCherry signal intensity inside the MΦs. Data are represented as the mean ± s.e.m. of *n* = 2 biological replicates, with 2-3 random fields of view analyzed per replicate. Flow cytometric analysis of (**E**) % of mCherry^+^ MΦs and (**F**) geometric mean of mCherry signal intensity of eGFP^+^ MΦs after co-culture with target cells. Data are represented as the mean ± s.e.m. of *n* = 6 biological replicates. Statistical significance was calculated with one-way ANOVA using multiple comparisons. ** indicates *p* < 0.01, and * indicates *p* < 0.03. Mock: untransduced CD34-derived MΦs; ctrl: control.

In a second approach, we analyzed cytokine secretion and antigen-specific phagocytic activity of CAR MΦs upon co-culture with either WT/mCherry^+^ or CEA^+^/mCherry^+^ HT1080 target cells. Similarly to THP-1-derived MΦ-like cells, primary CD34^+^ cell-derived CAR MΦs showed significantly enhanced IL-6 (Figure 5A) and TNFα (Figure 5B) secretion when co-cultured for four hours with CEA^+^/mCherry^+^ cells, as compared to control cultures with WT/mCherry^+^ cells as well as mock-transduced or eGFP control MΦs.

To investigate antigen-specific phagocytic activity of the CAR MΦs, confocal microscopy analyses were performed. As depicted in the upper panel of Figure 5C, low levels of the mCherry signal were detected inside eGFP ctrl MΦs as well as in CAR MΦs four hours after co-culture with WT/mCherry^+^ target cells. In contrast, when co-cultured with CEA^+^/mCherry^+^ target cells, both CAR constructs yielded an enhanced uptake of the mCherry signal in MΦs when compared to the eGFP control cells, with a higher phagocytic activity for CD3ζ CAR MΦs, as demonstrated by the higher percentage of yellow cells (Figure 5C, lower panel). To quantify the phagocytic activity of the CAR MΦs, we calculated the geometric mean of intracellular mCherry intensity observed by fluorescence intensity (Figure 5D). Data confirmed the similar background capacity of the different MΦ conditions to phagocytose WT/mCherry^+^ target cells. However, phagocytosis by anti-CEA-CAR MΦs was increased upon co-culture with CEA^+^/mCherry^+^ target cells. The strongest increase in mCherry signal intensity upon CEA^+^/mCherry^+^ co-culture was observed for CD3ζ CAR MΦs, while the increase for DAP12 CAR MΦs was less pronounced. 

Flow cytometric quantification of phagocytosis yielded similar results. No differences in the percentages of mCherry^+^ MΦs were detected after co-culture of MΦs with WT/mCherry^+^ target cells (Figure 5E). In contrast, co-culture with CEA^+^/mCherry^+^ target cells revealed a significant enhancement of mCherry^+^ cells in CD3ζ CAR MΦs as compared to DAP12 CAR MΦs and eGFP control MΦs (averages of 31.0% +/− 17.9%, 12.6% +/− 8.4%, and 7.1% +/− 3.9%, respectively). Again, we aimed to quantify phagocytic activity of our MΦs more stringently and calculated the geometric mean of intracellular mCherry intensity normalized to the eGFP control (Figure 5F). The CD3ζ CAR MΦs showed the strongest signal when co-cultured with CEA^+^/mCherry^+^ target cells, with a 2.3-fold increase in signal intensity compared to the eGFP control (ctrl). A significant difference was detected when co-culture of CD3ζ CAR MΦs with WT/mCherry^+^ or CEA^+^/mCherry^+^ target cells was compared, indicating the specific antigen-directed phagocytic activity of the anti-CEA CD3ζ CAR.

Taken together, we showed that CAR MΦs can be successfully generated from HSPCs without affecting their typical MΦs characteristics. Expression of both CARs resulted in an antigen-specific cytokine secretion pattern, and CAR MΦs that contained the CD3ζ signaling domain showed antigen-directed phagocytic activity against CEA^+^/mCherry^+^ target cells. 

## 4. Discussion

Although CAR T cell immunotherapy is producing impressive clinical results in patients with certain hematological cancers, the efficacy against solid tumors is still limited due to the immunosuppressive TME as well as fast T-cell exhaustion in this context [35]. Whereas numerous strategies to overcome these challenges are currently evaluated [36], alternative CAR approaches aim to target further types of immune cells, such as NK cells, DCs, or MΦs [6,37]. Among these, MΦs represent a promising cell type as they not only provide an important first-line of defense against invading pathogens, but are also an abundant cell type in the TME [10]. Properties such as phagocytic activity [8], antigen-presentation [9], and a great capacity to secrete pro-inflammatory cytokines [11] make MΦs valuable candidates to target tumor cells and counteract the immunosuppressive TME. In addition, MΦs are able to recruit and activate bystander immune cells to further enhance anti-tumor responses. Previous pre-clinical and clinical studies using macrophages as a therapeutic strategy to combat cancer have shown that the function of macrophages generated ex vivo is enhanced through pre-stimulation with LPS or IFNγ prior to adoptive transfer into the patient. On the basis of these results, we pre-stimulated macrophages with LPS [38,39].

Morrissey and colleagues have demonstrated a proof-of-concept for the CAR MΦ approach employing murine primary MΦs and showed enhanced phagocytosis of CD19^+^ lymphoma cells [20]. In addition, Niu and colleagues equipped a murine MΦ cell line with anti-CCR7 CARs designed to target an immunosuppressive subpopulation in the TME, which led to the inhibition of tumor growth and the prevention of metastasis in vivo [40]. Klichinsky and colleagues translated this strategy to human cells by transducing primary peripheral blood-derived monocytes using an adenovirus to overcome the resistance of MΦs to lentiviral transduction [21]. CAR MΦs were generated by differentiation of genetically modified monocytes and showed increased phagocytosis against CD19^+^ hematological malignancies as well as HER2^+^ and Mesothelin^+^ solid tumor cells. 

In our study, we investigated the potential of human CD34^+^ hematopoietic stem and progenitor cells (HSPCs) as an additional cell source to generate functional CAR MΦs. To generate sufficient amounts of CAR MΦs for subsequent analysis, we used HSPCs derived from cord blood, which have a high proliferative capacity and possess a primitive phenotype [41], rendering them susceptible for efficient lentiviral transduction. In principle, several sources of HSPCs are available for isolation, ex vivo manipulation, and potential re-transplantation. Besides bone marrow (BM) and CB, peripheral blood (PB), usually following G-CSF stimulation, can be used [18]. While BM samples require an invasive procedure and even general anesthesia for procurement of larger amounts of HSPCs, their isolation from G-CSF-mobilized PB is less invasive. However, a couple of weeks are needed for adequate HSPC mobilization, and trained personnel are required to perform apheresis. Furthermore, G-CSF treatment can cause side effects, such as bone pain, headache, and flu-like symptoms [42]. CB acquisition, on the other hand, requires no additional procedures to be performed on the patient. With respect to clinical application, CB can be HLA-typed, cryopreserved, and stored in CB banks until further use. Compared to BM or PB, the total yield of HSPCs after isolation is lower; however, CB-derived HSPCs have been shown to have a higher proliferative potential compared to their counterparts [43], which at least partially compensates for the lower yield after isolation. 

To test our hypothesis, we used an established protocol for isolation and lentiviral transduction of CB-derived CD34^+^ cells followed by effective differentiation into MΦs. For differentiation into MΦs, we used a cytokine cocktail of M-CSF/GM-CSF/IL-3 for 5 days and additional 5 days of M-CSF only for the final differentiation phase. This approach allowed generation of sufficient amounts of MΦs for our studies (≈2–4 × 10^6^ MΦs), but higher numbers are required for clinical purposes. To obtain clinically relevant cell numbers, extension of the expansion phase and an improvement of transduction efficiencies might be required. The generated MΦs showed a stable CAR expression during the differentiation process. Furthermore, the MΦs demonstrated a heterogeneous population expressing M1 and M2 markers, which might be sub-optimal for a clinical approach to treat cancer patients. However, our data indicate that despite the mixed polarity, the activated MΦs led to specific CEA^+^ HT1080 cell phagocytosis and showed an increased secretion of M1-associated cytokines, two important mechanisms to eradicate cancer cells. In order to analyze a potential enhancement of these mechanisms, MΦ pre-priming with M1-stimulating reagents such as GM-CSF or their addition during the differentiation process may be an alternative procedure. 

Several lines of evidence support the clinical translation of the CAR MΦ strategy outlined here. HSPC-derived CAR MΦs can be produced in clinical scale under suitable conditions using higher starting cell numbers of HSPCs. The in vivo persistence and stability of CAR MΦs have been shown in pre-clinical and clinical studies ([21]; NCT04660929). It is also of clinical interest to use frozen CAR MΦs as an off-the-shelf cell therapy product. In regard to GMP-compliant manufacturing, the CD34^+^ cell isolation process could be performed using the GMP-grade CliniMACS Prodigy system. Human serum or a platelet lysate, a growth factor-rich cell culture supplement derived from healthy donor human platelets, could be used to replace FBS. In order to avoid the sorting process, clinical-grade viral vectors with improved titers are expected to further improve transduction efficiencies of CD34^+^ cells. Existing protocols for expansion of other cell types will further produce higher numbers of CAR MΦs [44,45].

As a further advance to the CAR MΦ field, we focused on CEA as an exemplary solid tumor-associated antigen that is highly expressed in adenocarcinomas, in particular colorectal and pancreatic cancers [27]. Whereas CAR T cells [46,47] and CAR NK cells [43] showed promising results against CEA^+^ tumor cells, CEA has not been addressed as a target in the context of CAR MΦ therapy. We included two different signaling domains into our analyses: CD28-CD3ζ, which is already known to increase phagocytosis in CAR MΦs [20,21], and the endodomain of DAP12, a key accessory transmembrane protein known to play a crucial role in pro-inflammatory immune reactions in myeloid cells [48] and MΦ migration [49]. Furthermore, constitutive expression of DAP12 in monocyte-derived DCs led to an anti-tumor response, which resulted in reduction of tumor burden [50]. For both anti-CEA CAR constructs, expression was verified in two myeloid cell lines as well as THP-1-derived MΦ-like cells, and CAR expression did not alter cell phenotype or basic anti-bacterial function. Moreover, transduction of primary CB-derived human CD34^+^ cells resulted in efficient CAR expression, and subsequent differentiation into MΦs was achieved without adverse effects on cell phenotype, morphology, or basic anti-bacterial phagocytic activity.

Furthermore, we analyzed the capability of the generated CARs to enhance MΦ anti-tumor functionality against CEA^+^ cancer cells by two well-established mechanisms of action: phagocytosis and the secretion of pro-inflammatory cytokines. While THP-1-derived MΦ-like cells showed only a minor increase of phagocytosis of target cells, they displayed significantly enhanced pro-inflammatory cytokine secretion upon contact with CEA^+^ target cells, showing the antigen-specificity of the generated CAR constructs. Overall, the relatively low phagocytic ability of CAR and, in particular, DAP12-CAR expressing THP-1-derived MΦ-like cells may be attributed to the pre-mature monocytic developmental state of THP-1 cells, thus leading to inability to perform proper phagocytosis of the 10–15 µM large fibroblasts. In this context, an increased cytokine release might indicate an act of frustrated phagocytosis, i.e., the cells fail to phagocytose larger target cells, but are still able to secrete toxic agents for efficient killing [51].

In addition, in primary CD34^+^ cell-derived MΦs, we showed antigen specificity of anti-CEA CAR constructs and identified CD3ζ as a potent domain to induce both significantly-enhanced phagocytosis and an increased pro-inflammatory cytokine secretion upon contact to CEA^+^ HT1080 cells. In contrast, DAP12-expressing MΦs demonstrated enhanced cytokine secretion, but no improved phagocytic activity against target cancer cells. One possible explanation for these results is the number of ITAMs in the signaling domains. Previous CAR T cell studies have shown that a higher number of ITAMs in the signaling domain can enhance the CAR efficacy [52]. This might also apply to MΦs. ITAMs are intracellular motifs that act as docking sites for various cytoplasmic tyrosine kinases upon phosphorylation, which leads to further downstream signaling and cell activation [53]. Whereas the CD3ζ domain contains three ITAMs [31] as well as CD28 co-stimulation, the DAP12 domain has only one [54]. Another reason for the different activities of the two CAR MΦs may be related to the CAR expression levels. While average VCNs of 1 were determined for each construct in the respective CAR MΦs, the eGFP-sorted CD3ζ CAR MΦs showed a homogenous eGFP/CAR double-positive population in the flow cytometric analysis, but CAR expression was detected on half of the eGFP^+^ cells for DAP12 CAR MΦs. Here, it can be speculated that the DAP12 sequence affects the assembly of the CAR protein and, consequently, expression levels of this CAR or the accessibility of the extracellular spacer hinge region to the anti-IgG antibody. Incorporation of the native IgG1 hinge sequence into CAR constructs was shown to cause off-target CAR T-cell activation and activation of innate immune cells via binding to IgG gamma receptors (FcγRs) [55]. CAR MΦs were not activated when cultured in the absence of target cells, but our experimental designs did not include other FcR+ cell populations. However, incorporation of a modified hinge sequence, which was shown to maintain CAR T cell specificity with reduced off-target activity, may be an option to avoid potential problems in this respect.

As MΦs also function as antigen-presenting cells, there is a possibility that CAR MΦs might present the CAR or other cargo (e.g., fluorescent marker proteins such as eGFP) to the immune system and elicit immune responses. However, at least to our knowledge, evidence for this has not been reported in any pre-clinical or clinical studies of CAR MΦs. As with all novel therapeutic approaches, and especially those that are still early in development, potential cellular and humoral immune events against therapeutic cells need to be closely monitored.

In summary, our data revealed that human CB-derived CD34^+^ cells are a feasible cell source to generate CAR MΦs without the loss of beneficial cell characteristics. The engineered anti-CEA CAR MΦs displayed typical MΦ characteristics, showed antigen specificity, and secreted pro-inflammatory cytokines only upon contact to CEA^+^ target cells. In addition, CD3ζ CAR MΦs exhibited enhanced phagocytosis of CEA^+^ HT1080 cells. Having developed a novel CAR MΦ platform, a next step will be evaluation of the generated CAR MΦs in suitable tumor models. In this context, it might be interesting to analyze the potential of combined CAR MΦ and CAR T or CAR NK cell therapies. Such an approach seems therapeutically relevant, as Klichinsky and colleagues showed that CAR MΦs can counteract the immunosuppressive TME in a murine model by reprogramming tumor-associated macrophages (TAMs) to a pro-inflammatory phenotype and activating bystander T cells. IL-12 released from IL-12-TRUCK cells converted M2 macrophages to M1 macrophages for improved anti-tumor response [56]. Thus, the combination of CAR MΦs with CAR T or CAR NK therapies as effector cells that perform active tumor killing might be beneficial to further improve anti-tumor efficiency.

## Data Availability

Data is contained within the article.
